# Gene Polymorphisms and Susceptibility to Functional Dyspepsia: A Systematic Review and Meta-Analysis

**DOI:** 10.1155/2019/3420548

**Published:** 2019-04-15

**Authors:** Lijun Du, John J. Kim, Binrui Chen, Yawen Zhang, Hui Ren

**Affiliations:** ^1^Department of Gastroenterology, Sir Run Run Shaw Hospital, School of Medicine, Zhejiang University, Hangzhou, Zhejiang, China; ^2^Division of Gastroenterology & Hepatology, Loma Linda University Health, Loma Linda, CA 92354, USA; ^3^Ningbo City Medical Treatment Center Lihuili Hospital, Ningbo, Zhejiang, China

## Abstract

Functional dyspepsia (FD) is a common chronic gastrointestinal disorder with a complex, undefined mechanism. Clustering of patients with FD in families highlights the role of genetic factors in the pathogenesis of FD. We performed a systematic review and meta-analysis to clarify the associations between specific gene polymorphisms and FD susceptibility. PubMed, EMBASE, the Cochrane Library, and HuGE database were searched. An additive model was adopted to determine whether previous studied genes are associated with FD susceptibility. Carriers of minor allele in GNB3 825C>T (OR = 1.15, 95% CI 0.99-1.34, *P* = 0.07), SCL6A4 5HTTLPR (OR = 0.92, 95% CI 0.75-1.12, *P* = 0.40), and CCK-1R 779T>C (OR = 0.86, 95% CI 0.72-1.03, *P* = 0.09) genes failed to demonstrate susceptibility to FD. In a subgroup analysis, only minor allele (T) in GNB3 825C>T was associated with an increased susceptibility to the epigastric pain syndrome subtype (OR = 1.34, 95% CI 1.10-1.63, *P* = 0.003). Our meta-analysis based on available studies using an additive model failed to show that GNB3, SCL6A4, and CCK-1R polymorphisms are associated with FD susceptibility.

## 1. Introduction

Functional dyspepsia (FD) is a common gastrointestinal disorder affecting 20% of the global population [1]. FD is a complex, multifactorial disorder with possible etiologic factors including visceral hypersensitivity, brain-gut dysfunction, immune activation, *Helicobacter pylori* (*H. pylori*) infection, and delayed gastric emptying [2–6]. Patients with FD can be further categorized into epigastric pain syndrome (EPS) and postprandial distress syndrome (PDS) subtypes according to symptom characteristics [2].

Emerging studies demonstrate that susceptibility to FD is influenced by hereditary factors. Clustering of patients with FD in families highlights the role of genetic factors in the pathogenesis of FD [7, 8]. Furthermore, the presence of family history of abdominal pain or family history of indigestion increases the likelihood of developing FD [9]. Gene association studies to evaluate gene polymorphisms encoding neuromodulatory and immunomodulatory proteins related to gastrointestinal motility and visceral hypersensitivity, important in the pathogenesis of FD, have been performed [10]. Although previous studies identified several genes associated with FD susceptibility, the results are inconsistent. Therefore, we conducted a systematic review and meta-analysis to critically evaluate existing literature to determine whether specific genetic polymorphisms are associated with FD susceptibility and also stratified by EPS and PDS subtypes.

## 2. Materials and Methods

### 2.1. Literature Search

In order to search for all relevant studies investigating association of gene polymorphisms and FD susceptibility, we conducted a systematic literature search with PubMed, EMBASE, the Cochrane Library, and HuGE database through April 2018 using the following keywords and subject terms: “functional dyspepsia” or “dyspepsia” and “polymorphism,” “mutation,” or “variant.” Detailed information of search strategy can be found in the supplementary file (available here).

### 2.2. Eligibility Criteria

The inclusion criteria for studies were as follows: (1) case-control studies or cohort studies assessing the association between any gene polymorphism and FD, (2) sufficient data available to obtain genotypic frequencies to calculate odds ratio (OR) and 95% confidence interval (CI), and (3) studies in adult population. Non-English manuscripts, review articles, conference abstracts, or studies with insufficient demographic data were excluded.

### 2.3. Data Extraction

Two investigators (L.D., H.R.) independently extracted data. Data on the author, publication year, demographic characteristics, FD diagnostic criteria, genotyping method, and distribution of genotypes were collected. The quality of the studies was assessed by using the Newcastle-Ottawa Scale (NOS) based on three components: selection, comparability, and ascertainment of outcome [11]. From a range of 1 to 9 stars, studies with higher stars were considered to be higher quality. The values of the gene polymorphisms for those with at least three or more available studies were pooled to perform a meta-analysis designed a priori. An additive model that assumes the contribution of each allele to the relative risk was used to prevent multiple testing of differences between each pair of genotypes [12].

### 2.4. Statistical Analysis

The primary outcome was the OR of a specific gene polymorphism in patients with FD compared to the control population evaluated in three or more published studies. The presence of selection bias in control participants was evaluated by calculating the Hardy-Weinberg equilibrium (HWE), and genotypes frequencies of the control participants were compared using the chi-square test. ORs with 95% CI were calculated to assess the strength of the associations between gene polymorphisms and FD. When studies demonstrated significant heterogeneity, subgroup and sensitivity analyses were performed. Otherwise, a fixed effects model was used. Publication bias was evaluated by Begg's and Egger's tests. Two-sided *P* value <0.05 was considered significant. All statistical analyses were conducted using Stata 13.0 and Review manager 5.3.

## 3. Results

### 3.1. Literature Search and Study Characteristics

The initial literature search yielded 1,362 citations, of which 912 remained after removing duplicates. After screening titles and abstracts of studies, 768 were not relevant to the study aim and one was performed in the pediatric population. Furthermore, 70 review articles, 22 case reports, and 16 animal studies were excluded.

Finally, 35 case-control studies met the inclusion criteria for the systematic review. Thirteen studies evaluated the G-protein beta 3 subunit gene (GNB3) 825C>T in 1,390 FD and 3,058 control participants. Four studies evaluated the SCL6A4 serotonin transporter protein (5HTTLPR) in 326 FD and 1,285 control participants. Four studies evaluated the cholecystokinin receptor (CCK-1R) 779T>C in 521 FD and 677 control participants.

Other gene polymorphisms associated with the pathophysiology of FD have been studied in one (serotonin-1A (5HT1A), 5HT3A, 5HT4A, tumor necrosis factor-*α* (TNF-*α*), interleukin-10 (IL-10), IL-17, IL-1b, regulated upon activation normal T cell expressed and secreted (RANTES), sodium channel Na (SCN10A), neuronal nitric oxide synthase (nNOS), transient receptor potential vanilloid 1 receptor (TRPV1), cytochrome P450 (CYP1A), alpha 2A adrenergic receptor (ADRA2A), glutathione-S-transferases (GSTP1), CD14, catechol-o-methyltransferase (COMT), fatty acid amide hydrolase (FAAH), macrophage migration inhibitory factor (MIF), toll-like receptor-2 (TLR2), cyclooxygenase-1 (COX-1), ghrelin, and p22PHOX) or two (5HT2A) studies for susceptibility to FD [13–31].

**Figure 1:**
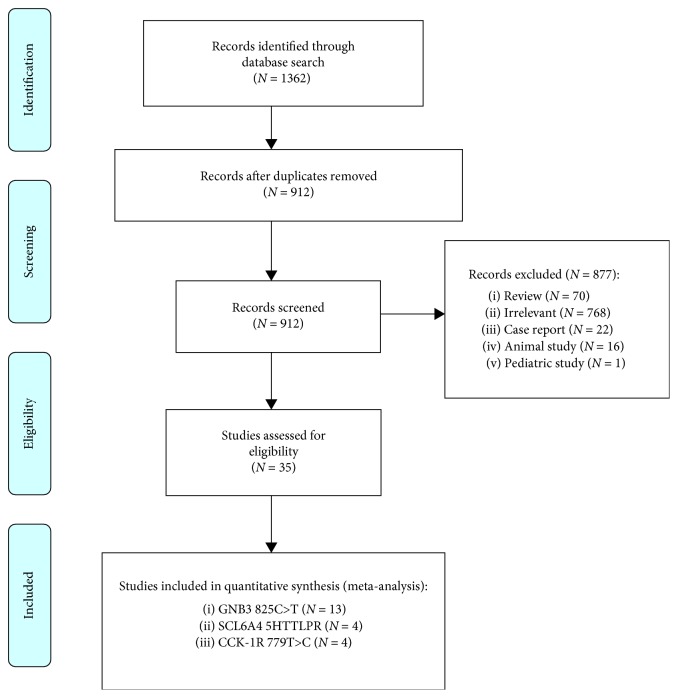
Flow diagram of selection process of eligible studies.

Meta-analysis was performed for the following three genes (GNB3 825C>T, SCL6A4 5HTTLPR, and CCK-1R 779T>C) with three or more studies available meeting the study criteria for meta-analysis. Quality scores of the selected studies ranged from seven to nine indicating moderate to high quality. The study selection process is summarized in Figure 1. Among the eligible studies, all were case-control studies. Only two studies demonstrated selection bias in the control group when calculating HWE [32, 33]. However, the association was not significantly changed when the two studies were excluded from the meta-analysis. Detailed characteristics of the studies included in the meta-analyses are shown (Table 1).

### 3.2. GNB3 825C>T Polymorphism and FD Susceptibility

**Figure 2:**
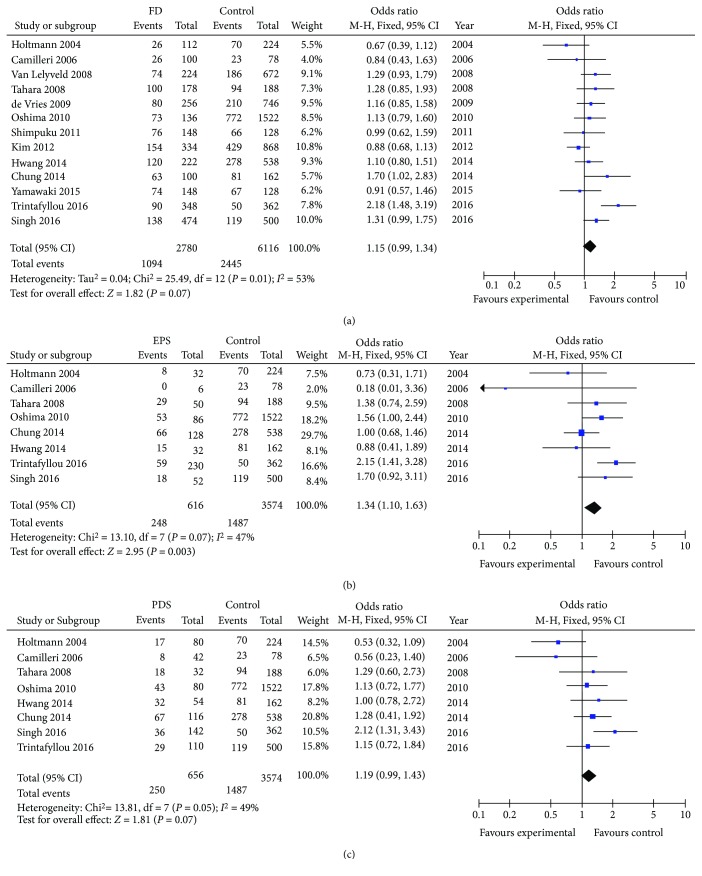
Forest plot of studies evaluating GNB3 825C>T and FD susceptibility using the additive genetic model.

The additive model was used to evaluate the association between GNB3 825C>T polymorphism and FD susceptibility (Figure 2, Table 2) [32–44]. Carriers of the minor allele (T) failed to demonstrate susceptibility to FD (OR = 1.15, 95% CI 0.99-1.34, *P* = 0.07). Given the presence of substantial heterogeneity (*I* ^2^ = 53%), the random effects model was utilized.

Subgroup analyses were further conducted by FD subtypes. Minor allele (T) was associated with increased susceptibility to the EPS subgroup (OR = 1.34, 95% CI 1.10-1.63, *P* = 0.003) and a trend towards increased susceptibility to the PDS subgroup (OR = 1.19, 95% CI 0.99-1.43, *P* = 0.07). Further subgroup analyses demonstrated that studies with *N* > 200 (OR = 1.22, 95% CI 1.01-1.49, *P* = 0.04) demonstrated increased susceptibility to FD but not in studies with sample size <200 (OR = 1.05, 95% CI 0.86-1.28, *P* = 0.65). Finally, no increased susceptibility was observed when the studies were stratified by studies from Asian (OR = 1.18, 95% CI 0.82-1.69, *P* = 0.38) or Western (OR = 1.10, 95% CI 0.97-1.25, *P* = 0.13) population.

In the sensitivity analysis removing one study at a time, no single study substantially influenced the pooled ORs. Similarly, there was little change in the estimated pooled ORs after excluding studies by Holtmann et al. [32] and Chung et al. [33], whose genotype distribution of the control group deviated from HWE. Finally, no evidence of publication bias was observed according to the Begg's test (Table 3).

### 3.3. SCL6A4 5HTTLPR Polymorphism and FD Susceptibility

**Figure 3:**
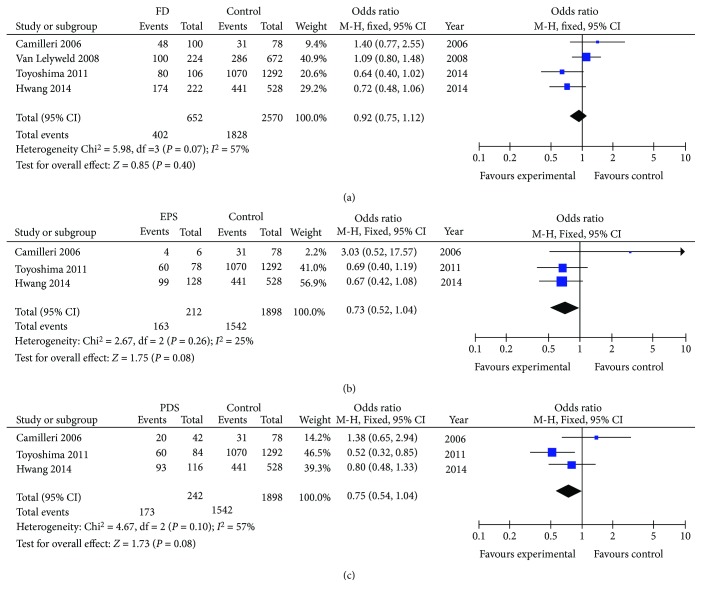
Forest plot of studies evaluating SCL6A4 5HTTLPR and FD susceptibility using the additive genetic model.

SCL6A4 5HTTLPR polymorphism as a predictor of FD susceptibility was examined (Figure 3, Table 2) [34, 35, 43, 45]. No association was observed between minor allele (S) and FD susceptibility (OR = 0.92, 95% CI 0.75-1.12, *P* = 0.40). Three studies were available for subgroup analysis evaluating susceptibility to FD subtypes. No association was observed in minor allele (S) and susceptibility to either FD subgroups: EPS (OR = 0.73, 95% CI 0.52-1.04, *P* = 0.08) or PDS (OR = 0.75, 95% CI 0.54-1.04, *P* = 0.08).

### 3.4. Other Gene Polymorphisms and FD Susceptibility

**Figure 4:**
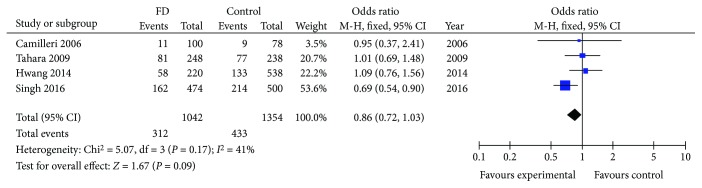
Forest plot of studies evaluating CCK-1R 779T>C and FD susceptibility using the additive genetic model.

CCK-1R 779T>C polymorphism failed to demonstrate susceptibility to FD (OR = 0.86, 95% CI 0.72-1.03, *P* = 0.09) (Figure 4 and Table 2) [34–36, 46]. Meta-analysis by FD subtype was not possible due to an insufficient number of studies.

In terms of individual studies, nNOS, CD14, MIF, and TRPV1 gene polymorphisms demonstrated increased susceptibility to FD [22, 42]. Ghrelin was associated with feeling of hunger in FD patients in one study [27]. Furthermore, a single study showed that p22PHOX, IL-1b-31CC genotype, and SCN10A were associated with decreased susceptibility to FD [16, 24, 28]. IL-10, IL-17, TNF-*α*, and 5HT3A were not associated with FD in a single study, respectively [16, 25, 36, 40]. Two studies exploring 5HT2A did not show any association [34, 40]. For studies evaluating FD subtypes, one study suggested that RANTES promoter -28G carrier was associated with a reduced susceptibility to PDS especially in patients with *H. pylori* infection [15]. COX-1 was associated with susceptibility to EPS in one study [31].

## 4. Discussion

In the present meta-analysis, we used additive genetic model to assess and measure the associations of the most extensively studied gene polymorphisms and susceptibility to FD. Carriers of the minor allele in genes GNB3 825C>T, SCL6A4 5HTTLPR, and CCK-1R 779T>C in FD failed to demonstrate susceptibility to FD. In the subgroup analysis, only minor allele (T) of GNB3 825C>T was associated with increased susceptibility to the EPS subtype.

Guanine nucleotide-binding proteins (G-proteins) play an integral role in the function of stimulus-response coupling of membrane receptors that are linked to intracellular effector system [47]. Hormones, neurotransmitters, and inflammatory stimuli involved in the pathophysiology of FD exert effect on cells probably by binding to G-protein-coupled receptors (GPCRs) [43]. GNB3 is the most widely studied G-protein in various disease processes including depression, cardiovascular disease, obesity, and irritable bowel syndrome [48, 49]. In FD, 825C>T variation-induced signal transduction contributes to the abnormalities in gastroduodenal sensory and motor functions in the setting of immune activation [32, 50]. Although the association of 825C>T and FD was initially reported in the Caucasian population [32], the results were not replicated in others. Furthermore, two meta-analyses were conducted to investigate the association between GNB3 polymorphism and susceptibility to FD but provided inconsistent results [51, 52]. Therefore, we performed an updated meta-analysis with additional studies and found that the carrier of the minor allele (T) in gene GNB3 825C>T was not associated with FD susceptibility. However, our results suggested GNB3 variation is associated with susceptibility to FD in patients with EPS (OR = 1.34, 95% CI 1.10-1.63, *P* = 0.003) and a trend towards susceptibility in PDS (OR = 1.19, 95% CI 0.99-1.43, *P* = 0.07) subtypes. Therefore, our result suggests that GNB3-mediated signal transduction is more closely linked to pain sensory rather than motility abnormalities. However, the observation of an increased susceptibility to FD in studies with sample size >200 but not in studies with sample size <200 suggested that heterogeneity present smaller studies may have impacted the effect estimate. Furthermore, the effect size of minor allele (T) on FD susceptibility in EPS subgroup was modest and should be interpreted with caution.

5-HT is the primary neurotransmitter involved in the regulation of psychological processes [53]. 5-HT plays a key role in the pathogenesis of both mood disorders and functional gastrointestinal disorders including FD [54, 55]. Psychiatric comorbidities including anxiety disorder and depression are more common in patients with FD compared to the control population [56]. Furthermore, a large body of neurobiological research have demonstrated that psychological factors impact gut physiology such as heightened pain sensitivity to gastric distention in patients with FD [57]. Clinical studies have also demonstrated that 5-HT3 receptor antagonism leads to relief of dyspeptic and anxiety symptoms [58, 59]. Given that 5-HT transporter (SERT) is the principle regulator of 5-HT levels by facilitating the reuptake, SERT gene-linked polymorphic region (5HTTLPR) may be involved in the pathogenesis of FD [60, 61]. More specifically, the short (S) allele has been associated with lower transcription and impaired reuptake of 5-HT compared to the long (L) allele [62]. However both overall and subgroup analyses in our study did not show that the carrier of S allele is associated with susceptibility to FD. However, bias affecting results are possible given that data on S allele status to conduct subgroup analysis was not consistently provided by the studies. Additional studies with larger sample size specifically evaluating S allele are needed to validate our findings.

Impaired gastric emptying is one of the major pathophysiologic mechanisms in FD. CCK-1, secreted by the neuroendocrine cells of the duodenal mucosa, has a physiologic function of delaying gastric emptying and inducing satiety. Furthermore, CCK-1 receptor belongs to the family of GPCRs and has been shown to be associated with symptoms of dyspepsia [63], and hyperresponsiveness to CCK has been demonstrated in patients with FD [64]. 779T carrier of CCK-1 is a predictor of PDS in Japanese male patients [46]. However, our meta-analysis failed to show an association between CCK-1 gene and susceptibility to FD.

Several other candidate genes have been explored for FD susceptibility. Genes that play important roles in the regulation of enteric primary afferents and brain-gut interaction (5-HT receptor genes, TRPV1, COMT, and SCN10), induction of inflammatory response (CD14, MIF, TNF-*α*, IL-17, IL-10, IL-1b, and RANTES), and mediation of gastric accommodation or relaxation (NOS) were hypothesized to be associated to FD susceptibility [24, 30, 65–70]. However, a meaningful meta-analysis was not able to be performed given the sparse number of studies. Furthermore, *H. pylori* is a potential confounder of FD. Previous studies have demonstrated that homozygous GNB3 825C>T may be associated with dyspeptic symptom among *H. pylori*-negative subjects [41]. However, the lack of data on *H. pylori* status in majority of the studies precluded a subgroup analysis to examine the association of genetic polymorphisms on FD susceptibility independent of *H. pylori* infection.

The present meta-analysis has limitations. First, the language of the manuscripts was restricted to English which may have excluded eligible studies in other languages. Furthermore, variable definition of dyspepsia, controls, and methodologies for measuring SNPs among studies led to study heterogeneity that may have affected the validity of the meta-analysis. In addition, the lack of data on long-term follow-up and treatment response precluded the assessment of the impact of gene polymorphisms on clinical application in FD. Finally, the lack of data on *H. pylori* status did not allow evaluation of genetic polymorphisms on FD susceptibility independent of *H. pylori* infection.

In conclusion, carriers of the minor allele in genes GNB3 825C>T, SCL6A4 5HTTLPR, and CCK-1R 779T>C were not associated with susceptibility to FD. In a subgroup analysis, only minor allele (T) of GNB3 825C>T was associated with an increased susceptibility to EPS subtype. The potential role of utilizing gene polymorphisms to decide diagnostic strategy and therapeutic interventions in FD is appealing, but robust evidence is lacking. Additional studies with larger sample size and detailed characterization of the patients are needed to clarify the role of genetic polymorphisms in FD.

## Figures and Tables

**Table 1:** Characteristics of studies included in the meta-analyses of GNB3 825C>T, SCL6A4 5HTTLPR, and CCK-1R 779T>C polymorphisms and FD susceptibility.

Author (year)	Country	Diagnostic criteria	Genotyping method	Age (year) (SD)		Gender (*n*) (M/F)		Genotype, CC/CT/TT, or LL/SL/SS (*n*)		HWE	NOS
				Case	Control	Case	Control	Case	Control		
GNB3 825C>T polymorphism											
Holtmann (2004)	Germany	Rome II	PCR	46.4 (1.9)	44.4 (1.3)	20/36	40/72	34/18/4	46/62/4	0.002	8
Camilleri (2006)	America	Rome II	PCR	55.8 (13.0)	59.5 (12.7)	27/23	17/22	32/10/8	17/21/1	0.07	7
Tahara (2008)	Japan	Rome II	PCR	60.1 (13.1)	61.1 (13.1)	33/56	55/39	20/38/31	23/48/23	0.84	7
Van Lelyveld (2008)	Netherlands	Rome II	PCR	42.3 (10.6)	41.9 (18.3)	32/80	94/242	48/54/10	180/126/30	0.25	9
De Vries (2009)	Netherlands	Rome II	PCR	49.7 (12.3)	40.1 (12.3)	62/66	120/253	60/56/12	199/138/36	0.10	7
Oshima (2010)	Japan	Rome III	PCR	44.5 (13.3)	48.0 (18.6)	25/43	325/436	17/29/22	191/368/202	0.37	8
Shimpuku (2011)	Japan	Rome III	PCR	59.2 (14.2)	37.2 (9.1)	36/38	57/7	14/44/16	17/28/19	0.32	9
Kim (2012)	Korea	Rome III	PCR	49.0 (15.0)	47.0 (15.0)	62/105	167/267	52/76/39	112/215/107	0.85	8
Chung (2014)	Korea	Rome III	PCR	46.8 (15.7)	50.5 (11.1)	21/29	41/40	5/27/18	15/51/15	0.02	8
Hwang (2014)	Korea	Rome III	PCR	50.3 (18.2)	53.3 (19.3)	35/77	112/157	20/62/29	64/132/73	0.77	7
Yamawaki (2015)	Japan	Rome III	PCR	N/A	N/A	39/35	34/30	14/46/14	16/29/19	0.46	9
Singh (2016)	India	Rome III	PCR-RFLP	38.4 (12.0)	37.3 (11.5)	173/64	152/43	125/86/26	143/95/12	0.45	9
Triantafyllou (2016)	Greece	Rome III	PCR-RFLP	50.2 (15.0)	53.7 (12.0)	61/113	68/113	99/60/15	137/38/6	0.11	7
SCL6A4 5HTTLPR polymorphism											
Camilleri (2006)	America	Rome II	PCR	55.8 (13.0)	59.5 (12.7)	27/23	17/22	14/24/12	14/19/6	0.91	7
Van Lelyveld (2008)	Netherlands	Rome II	PCR	42.3 (10.6)	41.9 (18.3)	32/80	94/242	37/50/25	108/170/58	0.52	9
Toyoshima (2011)	Japan	Rome III	PCR	51.8 (17.6)	46.8 (17.1)	26/27	326/320	3/20/30	24/174/448	0.17	7
Hwang (2014)	Korea	Rome III	PCR	50.3 (18.2)	53.3 (19.3)	35/77	112/157	3/42/66	8/71/185	0.71	7
CCK-1R 779T>C polymorphism											
Camilleri (2006)	America	Rome II	PCR	55.8 (13.0)	59.5 (12.7)	27/23	17/22	0/11/39	0/9/30	0.42	7
Tahara (2009)	Japan	Rome III	PCR-RFLP	58.0 (14.0)	59.6 (13.1)	57/67	71/48	12/57/55	11/55/53	0.54	7
Hwang (2014)	Korea	Rome III	PCR	50.3 (18.2)	53.3 (19.3)	35/77	112/157	4/50/56	13/107/149	0.26	7
Singh (2016)	India	Rome III	PCR-RFLP	38.4 (12.0)	37.3 (11.5)	173/64	152/43	19/124/94	46/122/82	0.96	9

M: male; F: female; HWE: Hardy-Weinberg equilibrium; NOS: Newcastle-Ottawa Scale; PCR: polymerase chain reaction; RFLP: restriction fragment length polymorphism; N/A: not available.

**Table 2:** Meta-analyses of GNB3 825C>T, SCL6A4 5HTTLPR, and CCK-1R 779T>C polymorphisms and FD susceptibility.

Group	Studies (*n*)	Case-control (*n*)	Additive model			
GNB3 825C>T			OR (95% CI)	*P* value	*I* ^2^ (%)	*P* value
Overall	13	1390/3058	1.15 (0.99-1.34)	0.07	52.9	0.01
Not deviate HWE	11	1223/2677	1.16 (1.04-1.29)	0.05	47.3	0.04
EPS	8	308/1787	1.34 (1.10-1.63)	0.003	46.6	0.07
HWE+	6	276/1594	1.44 (1.17-1.77)	0.001	46.8	0.09
PDS	8	328/1787	1.19 (0.99-1.43)	0.07	49.3	0.06
Not deviate HWE	6	261/1594	1.29 (1.03-1.62)	0.03	36.3	0.17
SCL6A4 5HTTLPR			OR (95% CI)	*P* value	*I* ^2^ (%)	*P* value
Overall	4	326/1285	0.92 (0.75-1.12)	0.40	57.0	0.07
EPS	3	106/949	0.73 (0.52-1.04)	0.08	25.2	0.26
PDS	3	121/949	0.75 (0.54-1.04)	0.08	57.2	0.10
CCK-1R 779T>C			OR (95% CI)	*P* value	*I* ^2^ (%)	*P* value
Overall	4	521/677	0.86 (0.72-1.03)	0.09	40.9	0.17

**Table 3:** Begg's and Egger's tests.

Association	Overall		EPS		PDS	
	*P*_1_ value	*P*_2_ value	*P*_1_ value	*P*_2_ value	*P*_1_ value	*P*_2_ value
GNB3 and FD risk	0.50	0.90	0.71	0.38	0.39	0.40
SCL6A4 and FD risk	0.73	0.85	0.30	0.08	0.30	0.22
CCK-1R and FD risk	0.73	0.47	N/A	N/A	N/A	N/A

*P*_1_ value: Begg's test; *P*_2_ value: Egger's test; EPS: epigastric pain syndrome; PDS: postprandial pain syndrome; N/A: not available.

## References

[1] Ford A. C., Marwaha A., Sood R., Moayyedi P. (2015). Global prevalence of, and risk factors for, uninvestigated dyspepsia: a meta-analysis. *Gut*.

[2] Sarnelli G., Vandenberghe J., Tack J. (2004). Visceral hypersensitivity in functional disorders of the upper gastrointestinal tract. *Digestive and Liver Disease*.

[3] Greydanus M. P., Vassallo M., Camilleri M., Nelson D. K., Hanson R. B., Thomforde G. M. (1991). Neurohormonal factors in functional dyspepsia: insights on pathophysiological mechanisms. *Gastroenterology*.

[4] Walker M. M., Talley N. J. (2017). The role of duodenal inflammation in functional dyspepsia. *Journal of Clinical Gastroenterology*.

[5] Talley N. J., Ford A. C. (2015). Functional dyspepsia. *New England Journal of Medicine*.

[6] Sugano K., Tack J., Kuipers E. J. (2015). Kyoto global consensus report on *Helicobacter pylori* gastritis. *Gut*.

[7] Shaib Y., El-Serag H. B. (2004). The prevalence and risk factors of functional dyspepsia in a multiethnic population in the United States. *The American Journal of Gastroenterology*.

[8] Locke G. R., Zinsmeister A. R., Talley N. J., Fett S. L., Melton L. J. (2000). Familial association in adults with functional gastrointestinal disorders. *Mayo Clinic Proceedings*.

[9] Gathaiya N., Locke G. R., Camilleri M., Schleck C. D., Zinsmeister A. R., Talley N. J. (2009). Novel associations with dyspepsia: a community-based study of familial aggregation, sleep dysfunction and somatization. *Neurogastroenterology & Motility*.

[10] Witte A. B., D'Amato M., Poulsen S. S. (2013). Duodenal epithelial transport in functional dyspepsia: role of serotonin. *World Journal of Gastrointestinal Pathophysiology*.

[11] Stang A. (2010). Critical evaluation of the Newcastle-Ottawa scale for the assessment of the quality of nonrandomized studies in meta-analyses. *European Journal of Epidemiology*.

[12] Gazouli M., Wouters M. M., Kapur-Pojskić L. (2016). Lessons learned — resolving the enigma of genetic factors in IBS. *Nature Reviews Gastroenterology & Hepatology*.

[13] Zhang Y., Li Y., Hao Z., Li X., Bo P., Gong W. (2016). Association of the serotonin receptor 3E gene as a functional variant in the microRNA-510 target site with diarrhea predominant irritable bowel syndrome in Chinese women. *Journal of Neurogastroenterology and Motility*.

[14] Tripathi S., Ghoshal U., Ghoshal U. C. (2008). Gastric carcinogenesis: possible role of polymorphisms of GSTM1, GSTT1, and GSTP1 genes. *Scandinavian Journal of Gastroenterology*.

[15] Tahara T., Shibata T., Yamashita H., Hirata I., Arisawa T. (2009). The role of RANTES promoter polymorphism in functional dyspepsia. *Journal of Clinical Biochemistry and Nutrition*.

[16] Tahara T., Shibata T., Okubo M. (2015). Association between interleukin-1*β* and tumor necrosis factor-*α* polymorphisms and symptoms of dyspepsia. *Molecular Medicine Reports*.

[17] Tahara T., Shibata T., Nakamura M. (2010). Homozygous TRPV1 315C influences the susceptibility to functional dyspepsia. *Journal of Clinical Gastroenterology*.

[18] Tahara T., Arisawa T., Shibata T. (2008). A genetic variant of the CD14 C-159T in patients with functional dyspepsia (FD) in Japanese subjects. *Journal of Clinical Biochemistry and Nutrition*.

[19] Tahara T., Arisawa T., Shibata T. (2008). Serotonin-2A receptor gene T102C polymorphism in patients with dyspeptic symptoms. *Hepatogastroenterology*.

[20] Tahara T., Arisawa T., Shibata T., Nakamura M., Wang F., Hirata I. (2008). COMT gene val158met polymorphism in patients with dyspeptic symptoms. *Hepatogastroenterology*.

[21] Singh R., Ghoshal U. C., Kumar S., Mittal B. (2017). Genetic variants of immune-related genes IL17F and IL10 are associated with functional dyspepsia: a case–control study. *Indian Journal of Gastroenterology*.

[22] Park J. M., Baeg M. K., Lim C. H., Cho Y. K., Choi M. G. (2014). Nitric oxide synthase gene polymorphisms in functional dyspepsia. *Digestive Diseases and Sciences*.

[23] Ghoshal U., Tripathi S., Kumar S. (2014). Genetic polymorphism of cytochrome P450 (CYP) 1A1, CYP1A2, and CYP2E1 genes modulate susceptibility to gastric cancer in patients with Helicobacter pylori infection. *Gastric Cancer*.

[24] Arisawa T., Tahara T., Shiroeda H. (2013). Genetic polymorphisms of SCN10A are associated with functional dyspepsia in Japanese subjects. *Journal of Gastroenterology*.

[25] Achyut B. R., Tripathi P., Ghoshal U. C., Moorchung N., Mittal B. (2008). Interleukin-10 (−819 C/T) and tumor necrosis factor-*α* (−308 G/A) gene variants influence gastritis and lymphoid follicle development. *Digestive Diseases and Sciences*.

[26] Camilleri M., Carlson P., McKinzie S. (2008). Genetic variation in endocannabinoid metabolism, gastrointestinal motility, and sensation. *American Journal of Physiology-Gastrointestinal and Liver Physiology*.

[27] Futagami S., Shimpuku M., Kawagoe T. (2013). The preproghrelin 3056 TT genotype is associated with the feeling of hunger and low acylated ghrelin levels in Japanese patients with Helicobacter pylori-negative functional dyspepsia. *Internal Medicine*.

[28] Tahara T., Shibata T., Wang F. (2009). A genetic variant of the p22PHOX component of NADPH oxidase C242T is associated with reduced risk of functional dyspepsia in Helicobacter pylori-infected Japanese individuals. *European Journal of Gastroenterology & Hepatology*.

[29] Tahara T., Shibata T., Wang F., Yamashita H., Hirata I., Arisawa T. (2010). Genetic polymorphisms of molecules associated with innate immune responses, TRL2 and MBL2 genes in Japanese subjects with functional dyspepsia. *Journal of Clinical Biochemistry and Nutrition*.

[30] Nakano H., Hirata I., Okubo M. (2007). Genetic polymorphisms of molecules associated with inflammation and immune response in Japanese subjects with functional dyspepsia. *International Journal of Molecular Medicine*.

[31] Arisawa T., Tahara T., Shibata T. (2008). Genetic polymorphisms of cyclooxygenase-1 (COX-1) are associated with functional dyspepsia in Japanese women. *Journal of Women's Health (2002)*.

[32] Holtmann G., Siffert W., Haag S. (2004). G-protein *β*3 subunit 825 CC genotype is associated with unexplained (functional) dyspepsia. *Gastroenterology*.

[33] Chung H. A., Lee S. Y., Lee H. J. (2014). G protein *β*3 subunit polymorphism and long-term prognosis of functional dyspepsia. *Gut and Liver*.

[34] Camilleri C. E., Carlson P. J., Camilleri M. (2006). A study of candidate genotypes associated with dyspepsia in a U.S. community. *The American Journal of Gastroenterology*.

[35] Hwang S. W., Kim N., Jung H. K. (2014). The association of *SLC6A4* 5-HTTLPR and *TRPV1* 945G>C with functional dyspepsia in Korea. *Journal of Gastroenterology and Hepatology*.

[36] Singh R., Mittal B., Ghoshal U. C. (2015). Functional dyspepsia is associated with GN*β*3 C825T and CCK-AR T/C polymorphism. *European Journal of Gastroenterology & Hepatology*.

[37] de Vries D. R., ter Linde J. J. M., van Herwaarden M. A., Smout A. J. P. M., Samsom M. (2009). Gastroesophageal reflux disease is associated with the C825T polymorphism in the G-protein *β*3 subunit gene (GNB3). *The American Journal of Gastroenterology*.

[38] Kim H. G., Lee K. J., Lim S. G., Jung J. Y., Cho S. W. (2012). G-protein Beta3 subunit C825T polymorphism in patients with overlap syndrome of functional dyspepsia and irritable bowel syndrome. *Journal of Neurogastroenterology and Motility*.

[39] Oshima T., Nakajima S., Yokoyama T. (2010). The G-protein *β*3 subunit 825 TT genotype is associated with epigastric pain syndrome-like dyspepsia. *BMC Medical Genetics*.

[40] Shimpuku M., Futagami S., Kawagoe T. (2011). G-protein *β*3 subunit 825CC genotype is associated with postprandial distress syndrome with impaired gastric emptying and with the feeling of hunger in Japanese. *Neurogastroenterology & Motility*.

[41] Tahara T., Arisawa T., Shibata T. (2008). Homozygous 825T allele of the GNB3 protein influences the susceptibility of Japanese to dyspepsia. *Digestive Diseases and Sciences*.

[42] Triantafyllou K., Kourikou A., Gazouli M., Karamanolis G. P., Dimitriadis G. D. (2017). Functional dyspepsia susceptibility is related to *CD14*, *GNB3*, *MIF*, and *TRPV1* gene polymorphisms in the Greek population. *Neurogastroenterology & Motility*.

[43] van Lelyveld N., Linde J. T., Schipper M., Samsom M. (2008). Candidate genotypes associated with functional dyspepsia. *Neurogastroenterology & Motility*.

[44] Yamawaki H., Futagami S., Shimpuku M. (2015). Leu72Met408 polymorphism of the ghrelin gene is associated with early phase of gastric emptying in the patients with functional dyspepsia in Japan. *Journal of Neurogastroenterology and Motility*.

[45] Toyoshima F., Oshima T., Nakajima S. (2011). Serotonin transporter gene polymorphism may be associated with functional dyspepsia in a Japanese population. *BMC Medical Genetics*.

[46] Tahara T., Arisawa T., Shibata T. (2009). 779 TC of CCK-1 intron 1 is associated with postprandial syndrome (PDS) in Japanese male subjects. *Hepatogastroenterology*.

[47] Kleuss C., Scherübl H., Hescheler J., Schultz G., Wittig B. (1992). Different *β*-subunits determine G-protein interaction with transmembrane receptors. *Nature*.

[48] Klenke S., Kussmann M., Siffert W. (2011). The GNB3 C825T polymorphism as a pharmacogenetic marker in the treatment of hypertension, obesity, and depression. *Pharmacogenetics and Genomics*.

[49] Zhu W., Li J., Sun X., Hua Q. (2017). Association of G-protein beta3 subunit gene C825T polymorphism with cardiac and cerebrovascular events in Chinese hypertensive patients. *Clinical and Experimental Hypertension*.

[50] Lindemann M., Virchow S., Ramann F. (2001). The G protein *β*3 subunit 825T allele is a genetic marker for enhanced T cell response. *FEBS Letters*.

[51] Dai F., Liu Y., Shi H. (2014). Association of genetic variants in *GNβ3* with functional dyspepsia: a meta-analysis. *Digestive Diseases and Sciences*.

[52] Song Y. Z., You H. Y., Zhu Z. H. (2016). The C825T polymorphism of the G-protein *β*3 gene as a risk factor for functional dyspepsia: a meta-analysis. *Gastroenterology Research and Practice*.

[53] Helton S. G., Lohoff F. W. (2015). Serotonin pathway polymorphisms and the treatment of major depressive disorder and anxiety disorders. *Pharmacogenomics*.

[54] Zangrossi H., Graeff F. G. (2014). Serotonin in anxiety and panic: contributions of the elevated T-maze. *Neuroscience and Biobehavioral Reviews*.

[55] Mohammadi M., Abdar H. T., Mollaei H. R., Hajghani H., Baneshi M. R., Hayatbakhsh M. M. (2017). Serotonin transporter gene (SLC6A4) polymorphism and mucosal serotonin levels in southeastern Iranian patients with irritable bowel syndrome. *Middle East Journal of Digestive Diseases*.

[56] Adibi P., Keshteli A. H., Daghaghzadeh H., Roohafza H., Pournaghshband N., Afshar H. (2016). Association of anxiety, depression, and psychological distress in people with and without functional dyspepsia. *Advanced Biomedical Research*.

[57] Drossman D. A., Dumitrascu D. L. (2006). Rome III: new standard for functional gastrointestinal disorders. *Journal of Gastrointestinal and Liver Diseases*.

[58] Talley N. J., van Zanten S. V., Saez L. R. (2001). A dose-ranging, placebo-controlled, randomized trial of alosetron in patients with functional dyspepsia. *Alimentary Pharmacology & Therapeutics*.

[59] Walstab J., Rappold G., Niesler B. (2010). 5-HT_3_ receptors: role in disease and target of drugs. *Pharmacology & Therapeutics*.

[60] Kulikov A. V., Gainetdinov R. R., Ponimaskin E., Kalueff A. V., Naumenko V. S., Popova N. K. (2018). Interplay between the key proteins of serotonin system in SSRI antidepressants efficacy. *Expert Opinion on Therapeutic Targets*.

[61] Heils A., Teufel A., Petri S. (1996). Allelic variation of human serotonin transporter gene expression. *Journal of Neurochemistry*.

[62] Murphy D. L., Lerner A., Rudnick G., Lesch K. P. (2004). Serotonin transporter: gene, genetic disorders, and pharmacogenetics. *Molecular Interventions*.

[63] Feinle C., Meier O., Otto B., D'Amato M., Fried M. (2001). Role of duodenal lipid and cholecystokinin A receptors in the pathophysiology of functional dyspepsia. *Gut*.

[64] Chua A. S. B., Keeling P. W. N., Dinan T. G. (2006). Role of cholecystokinin and central serotonergic receptors in functional dyspepsia. *World Journal of Gastroenterology*.

[65] Hansen M. B. (2003). Neurohumoral control of gastrointestinal motility. *Physiological Research*.

[66] Rietschel E. T., Kirikae T., Schade F. U. (1994). Bacterial endotoxin: molecular relationships of structure to activity and function. *The FASEB Journal*.

[67] Bloom B. R., Bennett B. (1966). Mechanism of a reaction in vitro associated with delayed-type hypersensitivity. *Science*.

[68] Kikuchi T., Kato K., Ohara S. (2000). The relationship between persistent secretion of RANTES and residual infiltration of eosinophils and memory T lymphocytes after *Helicobacter pylori* eradication. *The Journal of Pathology*.

[69] Tack J., Demedts I., Meulemans A., Schuurkes J., Janssens J. (2002). Role of nitric oxide in the gastric accommodation reflex and in meal induced satiety in humans. *Gut*.

[70] Drewes A. M., Schipper K. P., Dimcevski G. (2003). Gut pain and hyperalgesia induced by capsaicin: a human experimental model. *Pain*.

