# Supplementary material for: Gene Polymorphisms and Susceptibility to Functional Dyspepsia: A Systematic Review and Meta-Analysis
Source: Gastroenterol Res Pract. 2019 Apr 15;2019:3420548. doi: 10.1155/2019/3420548 (PMC6501140; doi:10.1155/2019/3420548)
Supplement: Supplementary Materials — A detailed search strategy is provided (using PubMed as functional dyspepsia an example). [file 3420548.f1.docx]

The detailed search strategy (using PubMed as an example) was listed below:

Search **(((((functional dyspepsia[Title/Abstract]) OR dyspepsia[Title/Abstract])) OR "Dyspepsia"[Mesh])) AND (((("Genetic Variation"[Mesh]) OR "Alleles"[Mesh]) OR "Polymorphism, Genetic"[Mesh]) OR ((((((polymorphism[Title/Abstract]) OR mutation[Title/Abstract]) OR variant[Title/Abstract]) OR variation[Title/Abstract]) OR gene[Title/Abstract]) OR allele[Title/Abstract]))**
